# Viable Severe Acute Respiratory Syndrome Coronavirus 2 Isolates Exhibit Higher Correlation With Rapid Antigen Assays Than Subgenomic RNA or Genomic RNA

**DOI:** 10.3389/fmicb.2021.718497

**Published:** 2021-11-12

**Authors:** Misbah Tariq, Dong-Min Kim, Choon-Mee Kim, Mi-Seon Bang, You Mi Lee, Jun-Won Seo, Da Young Kim, Na Ra Yun

**Affiliations:** ^1^Department of Internal Medicine, Chosun University College of Medicine, Gwangju, South Korea; ^2^Premedical Science, Chosun University College of Medicine, Gwangju, South Korea

**Keywords:** SARS-CoV-2, cell culture, infectivity, Ag-RDT, PCR, genomic RNA, subgenomic RNA

## Abstract

**Background:** Rapid identification and effective isolation are crucial for curbing the spread of severe acute respiratory syndrome coronavirus 2 (SARS-CoV-2). To meet this requirement, antigen-detection rapid diagnostic tests (Ag-RDTs) are essential.

**Methods:** Between February 2020 and August 2020 we performed a cohort study of patients with confirmed COVID-19. The clinical performance of Ag rapid fluorescence immunoassay (FIA) and Ag Gold was evaluated and compared in parallel with genomic and subgenomic real-time reverse transcription-polymerase chain reaction (rRT-PCR) and cell culture-based assays.

**Results:** In total, 150 samples were tested. Of these, 63 serial samples were obtained from 11 patients with SARS-CoV-2 and 87 from negative controls. Serial respiratory samples were obtained 2 days prior to symptom onset (-2) up to 25 days post-symptom onset. Overall, for rRT-PCR-positive samples (*n* = 51), the detection sensitivity of Ag rapid FIA and Ag Gold was 74.5% and 53.49%, respectively, with a specificity of 100%; however, for samples with low cycle threshold (Ct) values, Ag rapid FIA and Ag Gold exhibited a sensitivity of 82.61% (Ct ≤ 30, 5.6 log_10_RNA copies/mL) and 80% (Ct ≤ 25, 6.9 log_10_RNA copies/mL), respectively. Despite low analytical sensitivity, both Ag-RDTs detected 100% infection in cell culture-positive samples (*n* = 15) and were highly effective in distinguishing viable samples from those with subgenomic RNA (66.66%). For both Ag-RDTs, all samples that yielded discordant results (rRT-PCR + ve/Ag-RDT -ve) were also negative by culture.

**Conclusion:** The data suggest that Ag-RDTs reliably detect viable SARS-CoV-2; thus, they may serve as an important tool for rapid detection of potentially infectious individuals.

## Introduction

Since the first reported case in December 2019, the severe acute respiratory syndrome coronavirus 2 (SARS-CoV-2) pandemic has spread worldwide, causing enormous public health challenges. The spread of SARS-CoV-2 can be curbed by rapid detection, effective isolation, and tracing of their close contacts. The prevention and control strategies rely on a better understanding of duration of infectivity in proportion to the potential for transmission (Anderson et al., 2004).

To date, amplification of viral RNA via real time reverse transcription polymerase chain reaction (rRT-PCR) assay is considered a diagnostic reference standard method (Corman et al., 2020). While rRT-PCR is a highly sensitive diagnostic assay, it has certain limitations. This method is expensive, and being a laboratory-based assay, it often requires 24–48 h to obtain results in clinical practice (Larremore et al., 2021). Moreover, detection of viral RNA does not correlate with the detection of infectious virus by cell culture (Bullard et al., 2020; Wölfel et al., 2020); furthermore, a persistently positive rRT-PCR does not indicate whether the person is still contagious. Like other viruses, the RNA of SARS-CoV-2 can be detected beyond the period of infectivity (Falsey et al., 2003; Ip et al., 2016; Bullard et al., 2020). In a SARS-CoV-2 infected golden hamster model, the viral transmission window in respiratory samples was well associated with detection of viral infectiousness in cell lines but not with the presence of genomic RNA (Sia et al., 2020). Consequently, virological culture tends to be a more informative surrogate of viral infectiousness. The potential of the viral culture to guide infectivity is crucial in diagnostics; however, its usage is hampered by difficult procedures and the need for biosafety level 3 equipped facilities.

Recent surveillance revealed that with high-analytical but low-frequency assays such as real time PCR, numerous people were diagnosed with SARS-CoV-2 infection during the course of the disease when they were no longer contagious (Paltiel et al., 2020). Considering the diagnostic labor and duration of assays, a rapid, cost-effective, and relevant testing method is required for accurate detection of SARS-CoV-2 infection. A point-of-care test—antigen detection rapid diagnostic test (Ag-RDT)—can complement the screening tests if it effectively recognizes people who are spreading the virus. A recent report proposed that antigen assays may align better with culture-based tests compared to RT-PCR (Pekosz et al., 2020). The subgenomic RNA (sgRNA) indicates replicative intermediates of the viruses, rather than residual viral RNA (Perera et al., 2020). Moreover, the performance profiles of current FDA Emergency Use Authorization (EUA) SARS-CoV-2 antigen assays (Pekosz et al., 2020; Veritor, 2020) are optimal at time points and overlap with the temporal profile of sgRNA.

Furthermore, limited data are available to assess the performance of Ag-RDTs and their correlation with viral infectiousness (Veritor, 2020; Kohmer et al., 2021). The present study aimed to assess the clinical performance of rapid antigen tests in parallel with cell-culture and rRT-PCR (including genomic and subgenomic PCR)-based techniques to provide comprehensive correlation analysis with each diagnostic platform.

## Materials and Methods

### Diagnostic Criteria and Patients

A positive case was defined as a person testing positive for SARS-CoV-2 infection in compliance with diagnostic measures such as rRT-PCR and serological assays. rRT-PCR was performed by targeting in house designed N-gene as described below, and by using SD Biosensors kit (SD Biosensors, South Korea) targeting E and RdRP genes, as per the manufacturer’s protocol. The serological diagnosis was based on seroconversion or greater than fourfold increase in antibody titers, as described previously (Amanat et al., 2020; Hueston et al., 2020).

From February 2020 to August 2020, we recruited 11 patients who tested positive for SARS-CoV-2 infection, and thereafter, we performed analyses on serial respiratory samples. The samples were obtained from patients during their stay at Chosun University Hospital. To determine the specificity, samples were obtained from healthy individuals with no signs and symptoms of SARS-CoV-2 infection. The samples were collected and transported in a sample collection tube containing 3 mL of viral transport medium (VTM). All samples were preserved at −20°C and were used for viral RNA extraction and culture.

### Extraction of Viral RNA

A fully automated instrument (Bio-seam, South Korea) was used for extracting viral RNA using a Real-prep viral DNA/RNA kit (BioSewoom, South Korea). The extraction was performed with 200 μL of all samples as per the manufacturer’s protocol to get a final elution of 100 μL. Thereafter, the samples were stored at −80°C until further used for RT-PCR analysis.

### Detection of Severe Acute Respiratory Syndrome Coronavirus 2 RNA by One Step Quantitative Reverse Transcription-Polymerase Chain Reaction

One step RT-qPCR assay was performed to target the nucleocapsid (N) gene for detecting SARS-CoV-2. The primers and probe were designed in-house. Briefly, 5 μL of template was added to 4 μL of 5X RT-qPCR mixture (Roche), 0.5 μL of 200X RT enzyme solution (Roche), 1 μL (10 pmol/μL) of forward primer (nCov-NP-572F 5′-GCAACAGTTCAAGAAATTC-3′), 1 μL (10 pmol/μL) of reverse primer (nCov-NP-687R-5′-CTGGTTCAATCTGTCAAG- 3′), 1 μL (5 pmol/μL) of probe (nCov-NP-661P-5′-FAM-AAGCAAGAGCAGCATCACCG-BH Q1-3′), and 7.9 μL of RNAase free water to obtain a total reaction mixture of 20 μL. The analysis was performed in an Exicycler™ 96 (Ver. 4) Real-Time Quantitative Thermal Block (Bioneer, South Korea) under the following cycle conditions: 1 cycle at 50°C for 10 min and 95°C for 30 s followed by 45 cycles at 95°C for 5 s and 57°C for 30 s. SARS-CoV-2 sgRNAs were identified via RT-PCR as previously described (Wölfel et al., 2020).

The cycle threshold value (Ct-value) was analyzed using the Bioneer Package software, and the sample was considered positive if a visible amplification plot was observed at Ct ≤ 35 and negative with Ct > 35. We selected N-gene in order to determine the viral load. For this purpose, the Ct-values were converted to Log_10_ RNA copies/mL by utilizing the calibration curves as previously described (Wölfel et al., 2020). The results of other target genes including E-gene and RdRp along with N-gene are represented in Supplementary Table 1.

### Detection of Severe Acute Respiratory Syndrome Coronavirus 2 by Antigen-Detection Rapid Diagnostic Tests

The samples were tested with two lateral flow assays: PCL COVID-19 Ag Rapid FIA (fluorescence immunoassay) and PCL COVID-19 Ag Gold (PCL, Inc. South Korea); both are diagnostic medical devices that use a dual antibody sandwich reaction with an immunochromatographic assay to quantitatively detect the N-antigen of SARS-CoV-2 in human respiratory specimens. According to the manufacturer’s instructions, Ag Rapid FIA intended to identify SARS-CoV-2 antigen in human nasopharyngeal specimens, whereas Ag Gold detects SARS-CoV-2 antigen in human saliva or nasopharyngeal specimens. However, we used oropharyngeal, nasopharyngeal, and saliva samples for both assays, to allow parallel testing and comparison using different platforms. The recommended instructions for use according to the manufacturer include incorporation of the sample into the extraction buffer; however, we analyzed the samples in VTM, since it enabled rapid assessment of numerous previously characterized rRT-PCR clinical samples. Based on this approach, the manufacturer instructed the application of 100 μL of the sample immediately into the test card. Prior to testing, the samples were thawed and kept at room temperature. The samples were then vortexed and transferred into the test card well with an average incubation time of 15 min at room temperature. For Ag Rapid FIA, results were observed with the PCLOK EZ automated analyzer, in the quick test mode. Thereafter, the results of Ag Gold were read visually and recognized by two different individuals, who mutually decided the result. The complete assay was performed in a bio-safety level-2 facility with full personal protective equipment.

### Severe Acute Respiratory Syndrome Coronavirus 2 Cell Culture and Detection of Infectious Virus

All 63 rRT-PCRs characterized SARS-CoV-2 respiratory samples incubated in Vero E6 cells (Korean Cell Line Bank, KCLB no. 21587), using 24-well cell culture plates with glass coverslips. The infected cells were maintained in Dulbecco’s modified Eagle’s medium (DMEM, Gibco, Thermo Fisher Scientific, United States) supplemented with 2% fetal bovine serum and 1 × penicillin–streptomycin solution (Gibco, Thermo Fisher Scientific Inc., United States) and then cultured at 37°C under the presence of 5% CO_2_ for 3–5 days by daily observing the cytopathic effect (CPE). The results were characterized as negative if no CPE was observed within 5 days. Furthermore, viral RNA was extracted using the culture supernatant and analyzed via rRT-PCR at two passages to validate the proliferation of SARS-CoV-2. The completed assay was performed in bio-safety level-3 at Health and Environment Research Institute of Gwangju City.

### Statistical Analysis

The categorical variables were recorded as percentages and counts with Wilson score at 95% confidence intervals (CI), whereas the continuous variables were presented as mean, standard deviation (SD) or median, and interquartile range (IQR). The differences between means were compared using two sample *t*-tests. The McNemar test was used to analyze the test differences in dependent groups. The normality was evaluated using Kolmorgorov–Smirnov test. Moreover, sensitivity, specificity, positive predictive value (PPV), and negative predictive value (NPV) were calculated using two reference standards: (1) RT-PCR (N-gene) to confirm clinical specimens for diagnosis and (2) SARS-CoV-2 culture in cell line to identify infectiousness. The inter-rater agreement between rRT-PCR and Ag-RDTs was calculated using Cohen’s weighted kappa (*K*-value) (Cohen, 1968). The interpretations of *K*-value were characterized as follows: < 0.20 as poor; 0.21–0.40 as fair; 0.41–0.60 as moderate; 0.61–0.80 as substantial; and > 0.8 as almost prefect agreement (Landis and Koch, 1977). To determine the accuracy of the assay, the receiver operating characteristic (ROC) curve was generated, and area under the ROC curve (AUC) was observed (Hajian-Tilaki, 2013). All data were analyzed using MedCalc statistical software (Ostend, Belgium), and the *p-*values were reported as two tailed with < 0.05 indicating statistical significance.

### Study Approval

The study was approved by the Institutional Review Board (IRB) of Chosun University Hospital (CHOSUN 2020-04-003-002). Written informed consent was obtained from all participants.

## Results

In total, 150 samples were tested; of these, 63 serial samples were obtained from SARS-CoV-2 positive patients [oropharyngeal swab (*n* = 10), nasopharyngeal swab (*n* = 26), and saliva (*n* = 27)] and 87 samples from healthy individuals as negative controls. All 63 samples were tested via rRT-PCR, cell culture, and Ag rapid FIA; however, only 54 samples were available for Ag Gold analysis. The samples included in the present study were obtained 2 days prior to the symptom onset (-2) up to 25 days post-symptom onset (PSO). Most samples were collected during the early stage of disease course with median duration of 1-day PSO (IQR; −1.25 to 5.25). Of the 63 samples, 51 (80.59%) were RT-PCR positive with mean Ct-value of 26.52 (± 4.74; range, 16.56–34.47) equivalent to 6.5 log_10_ RNA copies/mL (range, 9.28–4.41 log_10_ RNA copies/mL).

### Identification of Viral Infectiousness in Cell Line

**FIGURE 1:**
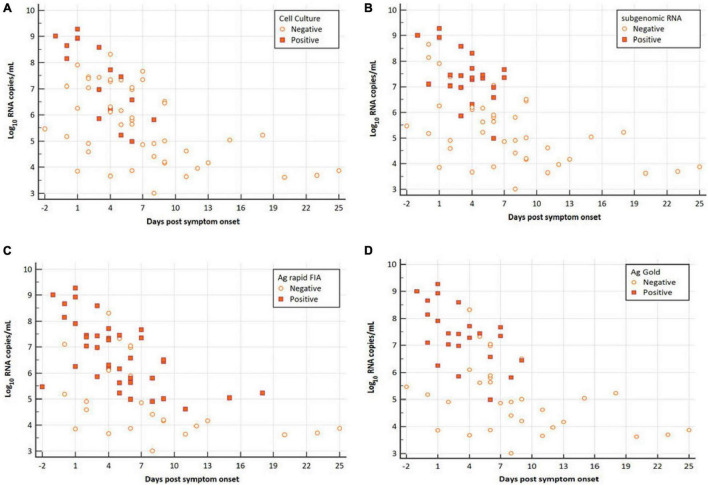
Viral RNA loads (Log_10_ RNA copies/mL, N-gene) in respiratory samples vs. the days post-symptom onset. **(A)** SARS-CoV-2 Vero E6 cell culture, **(B)** subgenomic RNA, **(C)** Ag rapid FIA, and **(D)** Ag Gold. Orange boxes represent positive samples, and open circles represent negative samples.

Among all patients with SARS-CoV-2 infection, 63 samples were analyzed for the presence of infectious virus using Vero E6 cells; moreover, the viral RNA load was determined via RT-qPCR (Figure 1). Infectious SARS-CoV-2 was successfully cultivated from 15 respiratory tract samples (23.80%) from 6 patients (54.54%). The mean viral load of culture positive samples was significantly higher than that of culture negative samples (7.29 vs. 5.65 log_10_ RNA copies/mL, *p* = 0.0003; Table 1). A significant difference was observed in mean day PSO of culture positive and negative samples (3.20 vs. 6.87, *p* = 0.020; Table 1).

**TABLE 1:** Comparison of clinical parameters for virological cell culture, subgenomic RNA PCR, Ag rapid FIA, and Ag Gold.

Assays and parameters		Positive	Negative	*p*-value*
**Cell culture**		15	48	
Viral RNA load (log_10_ RNA copies/mL)	Mean	7.29	5.65	0.0003
	95% CI (%)	6.48–8.10	5.23–6.07	
	SD	1.45	1.44	
Days PSO	Mean	3.20	6.87	0.020
	95% CI (%)	1.76–4.63	5.19–8.55	
	SD	2.59	5.77	
**Subgenomic RNA**		22	41	
Viral RNA load (log_10_ RNA copies/mL)	Mean	7.40	5.31	<0.0001
	95% CI (%)	6.95–7.85	4.88–5.74	
	SD	1.01	1.35	
Days PSO	Mean	3.59	7.29	0.009
	95% CI (%)	2.62–4.55	5.34–9.23	
	SD	2.17	6.16	
**Ag rapid FIA**		38	25	
Viral RNA load (log_10_ RNA copies/mL)	Mean	6.72	5.01	<0.0001
	95% CI (%)	6.29–7.14	4.40–5.63	
	SD	1.28	1.49	
Days PSO	Mean	7.76	4.84	0.0351
	95% CI (%)	5.02–10.49	3.48–6.19	
	SD	6.63	4.12	
**Ag Gold**		23	31	
Viral RNA load (log_10_ RNA copies/mL)	Mean	7.38	5.04	<0.0001
	95% CI (%)	6.91–7.86	4.58–5.51	
	SD	1.09	1.27	
Days PSO	Mean	3.26	8.54	<0.0005
	95% CI (%)	2.03–4.48	6.22–10.86	
	SD	2.83	6.23	

*Viral RNA loads are expressed as log_10_ RNA copies/mL.*

*CI, confidence interval; SD, standard deviation; PSO, post symptom onset; FIA, fluorescent immunoassay.*

*Two-sample t-test (two-tailed).

*In the case of cell culture, subgenomic RNA, and Ag rapid FIA, the data set includes results from 63 serial respiratory samples obtained from SARS-CoV-2 positive subjects, while 54 samples were analyzed for Ag Gold assay.*

### Antigen-Detection Rapid Diagnostic Test Performance in Correlation With Real-Time Reverse Transcription-Polymerase Chain Reaction

**FIGURE 2:**
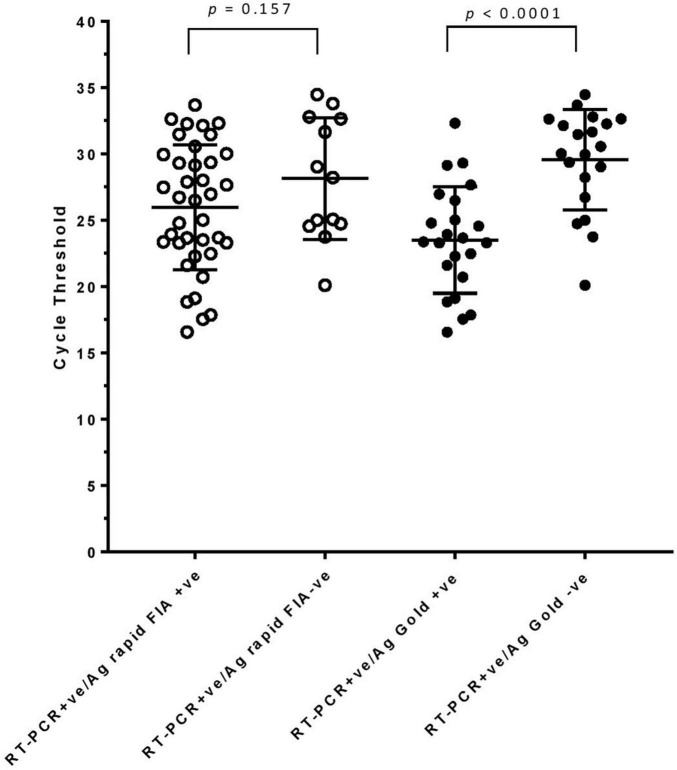
rRT-PCR cycle threshold values (N-gene) in samples testing either Ag-RDT positive or negative. Mean (standard deviation) with *p*-values are depicted.

**FIGURE 3:**
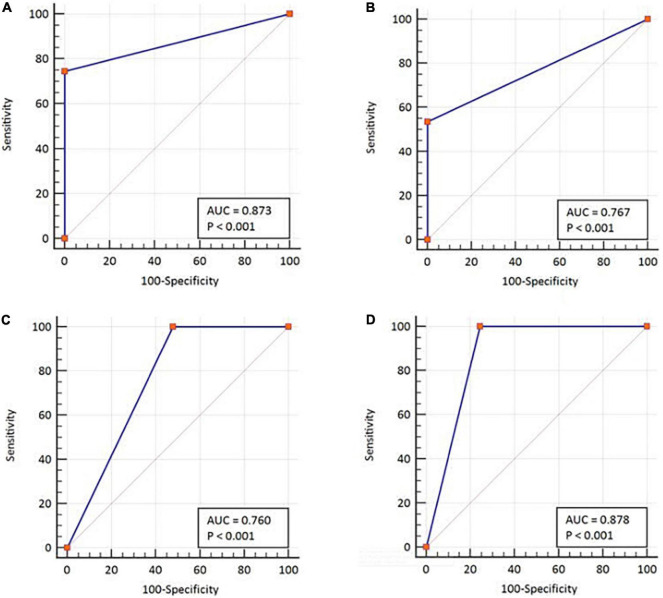
ROC-curve analysis with respect to the two diagnostic classification variables. By using rRT-PCR as a reference, **(A)** Ag rapid FIA and **(B)** Ag Gold. Using cell culture test as a reference, **(C)** Ag rapid FIA and **(D)** Ag Gold. ROC, receiver operating characteristic; AUC, area under the ROC curve. The AUCs were characterized as follows: 0.5 as no discrimination; 0.7–0.8 as excellent; and >0.9 was considered outstanding.

In parallel, the samples were subjected to Ag-RDTs using two different assays. Overall, for rRT-PCR positive samples, the detection sensitivity of Ag rapid FIA (*n* = 51) and Ag Gold (*n* = 43) was 74.51% (38/51, 95% CI; 60.4–85.7) and 53.49% (23/43, 95% CI; 37.7–68.8), respectively. Both assays were performed with 100% specificity for rRT-PCR negative samples (*n* = 99 and *n* = 98). Discordant results (rRT-PCR +ve/Ag-RDT -ve) were observed in 13 samples for Ag rapid FIA and in 20 samples for Ag Gold (Figure 2). Moreover, concordance between Ag-RDTs and rRT-PCR exhibited a kappa value of 0.79 for Ag rapid FIA and 0.61 for Ag Gold, thereby indicating a substantial and moderate agreement (Table 2). The ROC-curve analysis revealed an AUC of 0.837 (95% CI; 0.808–0.921) and 0.767 (95% CI; 0.689–0.834), respectively (*p* < 0.001) (Figure 3). According to the range of mean Ct-values (≤20, >20– ≤25, > 25– ≤ 30, >30– ≤ 35, >35), both antigen assays revealed 100% detection rate (95% CI; 47.82–100) for samples at Ct < 20. A noticeable decrease in the detection rate of Ag Gold was observed at Ct > 25, with overall sensitivity of 80% (95% CI; 56.34–94.27) at Ct ≤ 25; however, Ag rapid FIA sustained detection rate of 82.61% (95% CI; 68.58–92.18) until Ct ≤ 30. No detection was observed at Ct > 35 for both Ag-RDTs (Table 3). Furthermore, in terms of days PSO, the sensitivities of Ag-RDTs are higher at the initial stage of the disease course, followed by a progressive decline in further ranks (Table 4). The difference was statistically significant between mean viral loads and day PSO of Ag-RDT positive and negative samples (Table 1).

**TABLE 2:** Evaluation of interrater agreement between two types of antigen test and subgenomic RNA with rRT-PCR (N-gene) and cell culture-based techniques.

rRT-PCR	Ag rapid FIA			Ag Gold			subgenomic RNA		
	Positive	Negative	Row marginal	Positive	Negative	Row marginal	Positive	Negative	Row marginal
Positive	38	13	51 (34.0%)	23	20	43 (30.5%)	22	29	51 (81.0%)
Negative	0	99	99 (66.0%)	0	98	98 (69.5%)	0	12	12 (19.0%)
Column marginal	38 (25.3%)	112 (74.7%)		23 (16.3%)	118 (83.7%)		22 (34.9%)	41 (65.1%)	
Weighted kappa	0.79			0.61			0.22		
Standard error	0.05			0.07			0.06		
95% CI	0.68–0.89			0.47–0.75			0.09–0.35		
**Cell culture**									
Positive	15	0	15 (23.8%)	13	0	13 (24.1%)	10	5	15 (23.8%)
Negative	23	25	48 (76.2%)	10	31	41 (75.9%)	12	36	48 (76.2%)
Column marginal	38 (60.3%)	25 (39.7%)		23 (42.6%)	31 (57.4%)		22 (34.9%)	41 (65.1%)	
Weighted kappa	0.34			0.59			0.35		
Standard error	0.08			0.10			0.12		
95% CI	0.17–0.50			0.39–0.80			0.11–0.60		

*rRT-PCR, real time reverse transcription polymerase chain reaction; CI, confidence interval.*

*The interpretations of K-value were characterized as follows: < 0.20 as poor; 0.21–0.40 as fair; 0.41–0.60 as moderate; 0.61–0.80 as substantial and > 0.8 is considered almost perfect agreement.*

**TABLE 3:** Sensitivity of Ag rapid FIA and Ag Gold assays determined by SARS-CoV-2 N gene rRT-PCR cycle threshold (Ct) value.

Ct-value	*N*	Positive	Negative	Sensitivity (%)	95% CI (%)
**Ag rapid FIA**					
≤20	5	5	0	100.00%	47.82–100
>20–≤25	17	12	5	70.59%	44.04–89.69
>25–≤30	16	13	3	81.25%	54.35–95.95
>30–≤35	13	8	5	61.54%	31.58–86.14
>35	12	0	12	0.00%	0.00–26.46
**Ag Gold**					
≤20	5	5	0	100.00%	47.82–100
>20– ≤ 25	15	11	4	73.33%	44.90–92.21
>25– ≤ 30	12	6	6	50%	21.09–78.91
>30– ≤ 35	11	1	10	9.09%	0.23–41.28
>35	11	0	11	0%	0.00–28.49

*rRT-PCR, real time reverse transcription polymerase chain reaction; CI, confidence interval; N, number of total samples.*

**TABLE 4:** Sensitivity of Ag rapid FIA and Ag Gold determined by days post-symptom onset.

Days post-symptom onset	Ct-value (mean ± SD)	*N*	Positive	Negative	Sensitivity (%)	95% CI (%)
**Ag rapid FIA**						
−2–3	24.94 ± 5.72	21	15	6	71.43%	47.82–88.72
4–7	27.47 ± 4.45	24	15	9	62.50%	40.59–81.20
8–14	33.41 ± 3.78	13	6	7	46.15%	19.22–74.87
>14	34.9 ± 2.86	5	2	3	40.00%	5.27–85.34
**Ag Gold**						
−2–3	24.52 ± 5.80	18	14	4	77.78%	52.36–93.59
4–7	27.41 ± 4.86	19	7	12	36.84%	16.29–61.64
8–14	33.25 ± 3.90	12	2	10	16.67%	2.09–48.41
>14	34.90 ± 2.86	5	0	5	0.00%	0.00–52.18

### Antigen-Detection Rapid Diagnostic Tests Performance in Correlation With *in vitro* Infection and Subgenomic RNA

**FIGURE 4:**
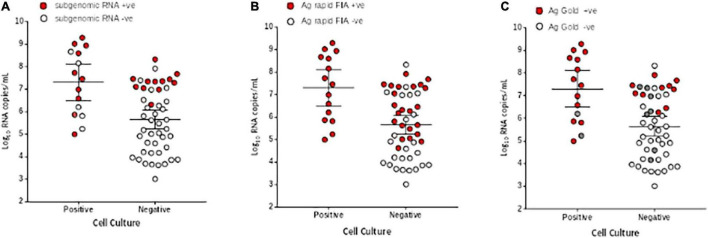
Comparison of viral RNA loads (Log_10_ RNA copies/mL) in respiratory samples of culture positive and negative samples. **(A)** Subgenomic RNA, **(B)** Ag rapid FIA, and **(C)** Ag Gold. The respiratory specimens are plotted by log_10_ RNA copies/mL (y-axis) and are stratified by SARS-CoV-2 cell culture results (positive, *n* = 15, negative, *n* = 48). Subgenomic RNA, Ag rapid FIA, and Ag Gold positive results are indicated as red data points (*n* = 25, 38, and 23, respectively), whereas negative (*n* = 41,22, and 31 respectively) as white data points. In case of Ag Gold, the unavailability of samples is indicated as grey data points. The horizontal lines on x-axis indicate mean and 95% confidence interval.

We further evaluated the performance of Ag-RDTs with respect to infectious SARS-CoV-2 samples and sgRNA. The analysis revealed that both Ag rapid FIA and Ag Gold detected 100% (95% CI; 78.20–100 and 75.29–100) of the infectious samples (15/15 and 13/13) and showed better performance in distinguishing infectious samples compared to sgRNA PCR assay [66.66% (10/15), *p* = 0.06 and 0.25] (Figure 4). The ROC-curve analysis revealed an AUC of 0.760 (95% CI; 0.636–0.859) and 0.878 (95% CI; 0.760–0.951) for Ag rapid FIA and Ag Gold, respectively (*p* < 0.001) (Figure 3). Only 29.4% (15/51) of rRT-PCR positive samples were infectious when tested by cell culture infectivity. In contrast, both Ag-RDTs demonstrated better correlation with cell culture infectivity [39.47% (15/38, *p* = 0.322) and 56.52% (13/23, *p* = 0.027)]. For both Ag-RDTs, all these samples yielded discordant results (rRT-PCR +ve/Ag-RDT -ve) and were detected as negative on culturing. Concordance between Ag-RDTs and cell culture demonstrated kappa values of 0.34 and 0.59, indicating a fair and moderate agreement (Table 2). The kappa value between sgRNA and cell culture was 0.35, indicating fair agreement (Table 2). Furthermore, the data suggest that detection of sgRNA is not well correlated with that of infectious virus in Vero E6 cells and was predicted poorly if cell cultures were positive (PPV of 47.62%, 95% CI, 32.62–63.06) (Figure 4 and Supplementary Figure 2).

### Probit Analysis

**FIGURE 5:**
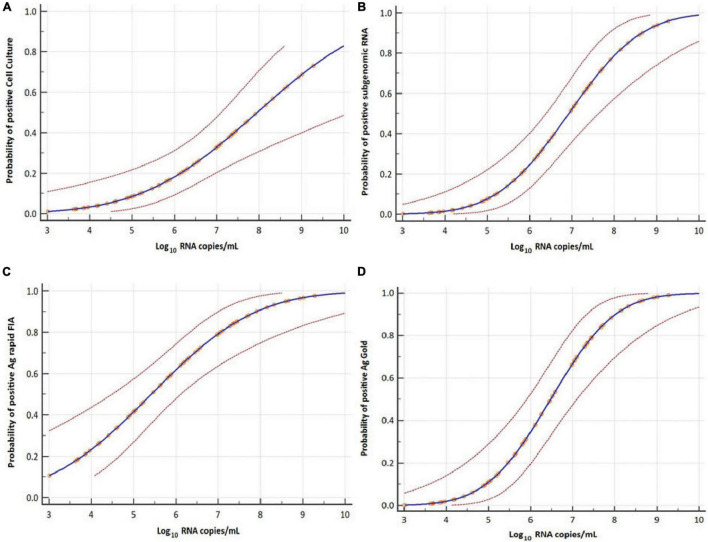
Probit analyses for detecting infectious virus in respiratory samples. Cell culture **(A)**, subgenomic RNA **(B),** Ag rapid FIA **(C)**, and Ag Gold **(D)** with respect to viral RNA load in Log_10_ copies per mL (N-gene). Blue line represents the probit curve, and dotted red lines represent the 95% confidence interval. Circles indicate marker points.

**FIGURE 6:**
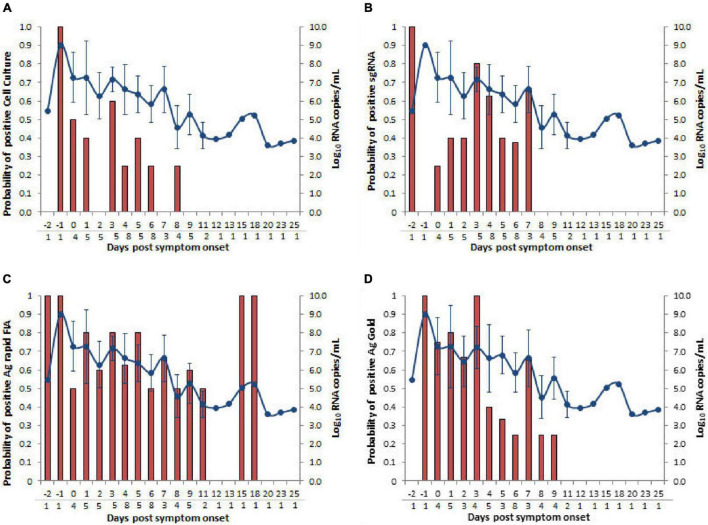
Comparison of days post-symptom onset to the probability of positive and SARS-CoV-2 N gene viral load RNA copies/mL. Four types of assays, **(A)** cell culture on Vero cells **(B)** subgenomic RNA (sgRNA), **(C)** Ag rapid FIA, and **(D)** Ag Gold were analyzed. Viral loads are represented by the line graph with circles. Probability of positive is depicted by the bar graph. The numbers of samples tested according to days post-symptom onset are presented below the gray line on the *x*-axis.

The probit analysis was performed for each diagnostic method with respect to viral load RNA copies/mL obtained from rRT-PCR (N-gene) and day PSO (Figures 3, 5). The analysis revealed < 5% probability of isolating infectious SARS-CoV-2 when the viral load was below 4.41 log_10_ RNA copies/mL (95% CI, 0.95–5.48; Ct = 34.347) (Figure 5A). Moreover, positive growth was observed until Day 8 PSO at 4.99 log_10_ RNA copies/mL (Ct = 32.31; Figure 6A) with less than < 5% probability of isolating infectious SARS-CoV-2 at Day 11.7 PSO (95% CI 8.77–14.68, Supplementary Figure 1A) or below. The sgRNA yielded a positive result at a rate of 5% for a viral load of 4.71 log_10_ RNA copies/mL, whereas the Ag rapid FIA and Ag Gold corresponded to a positive result of 5% at viral loads of 2.26 and 4.49 log_10_ RNA copies/mL, respectively (Figures 5B–D). The probability of 5% detection was observed until Days 15.01, 34.60, and 13.56 PSO for sgRNA, Ag rapid FIA, and Ag Gold, respectively (Supplementary Figure 1). The Ag rapid FIA revealed the probability of positive results until 18 days PSO (Figure 6C); consequently, the probit analysis exhibited probability of detecting more days than other assays. Furthermore, the probability of positive results over the complete range of days PSO is illustrated in Figure 6.

## Discussion

The present study demonstrates the performance characteristics of the Ag-RDTs for detecting SARS-CoV-2 in respiratory samples and describes the correlation between rRT-PCR and viable SARS-CoV-2. Our analytical findings revealed that both Ag-RDTs are comparable and were performed with a high sensitivity (100%) for detecting viable SARS-CoV-2 in respiratory samples. The overall sensitivity was lower (53.49–74.51%) for that of rRT-PCR. However, revaluation of samples with high viral loads, indicating early course of infection (Bullard et al., 2020; La Scola et al., 2020), revealed good correlation (around 80%). Even though the correlation between transmissibility and viral load remains unclear, several studies have reported that samples with higher viral load of ≥ 6 log_10_ RNA copies/mL would be associated with infectivity in cell culture (La Scola et al., 2020; Perera et al., 2020; van Kampen et al., 2020; Wölfel et al., 2020). Consistent with the findings of other studies, our results suggest that Ag-RDTs, although less sensitive, align efficiently with the cell culture-based techniques to identify infectiousness than rRT-PCRs (Pekosz et al., 2020; Perera et al., 2020; Toptan et al., 2020; Kohmer et al., 2021).

The key aspect of utilizing a point-of-care test is its ability to discriminate between non-infected and infected individuals who can potentially transmit the virus. Our correlation analysis revealed that both Ag-RDTs effectively identified all SARS-CoV-2 viable specimens (100%) that were positive in the cell culture. Notably, almost 26% (4/15) of the samples, despite having a relatively low viral load (<6 log_10_ RNA copies/mL), still tested positive in cell culture (Figure 4). Even though less sensitivity of Ag-RDTs was observed for low viral loads (<6 log_10_ RNA copies/mL), these culture positive samples also tested positive with Ag-RDTs but not with sgRNA PCR assay. Although no direct evidence indicates that virus infectivity in the cell culture correlates with virus transmission in humans, a correlation was observed between the virus detection and communicable period in a golden Syrian animal model (Sia et al., 2020); this is considered an indicator of infectivity. In the present study, Ag rapid FIA met the minimum performance requirement of WHO, which supports the use of SARS-CoV-2 Ag-RDTs with > 80% sensitivity and ≥ 97% specificity at a viral load of > 6 log_10_ RNA copies/mL (equivalent to Ct ≤ 28; N-gene) using the reference method of nucleic acid amplification (WHO, 2021).

Moreover, our analysis revealed that presence of viral sgRNA was not associated with infection in the cell culture. Furthermore, one study (Wölfel et al., 2020) reported that the presence of sgRNA indicates active viral replication, and thus viral infection; however, two recent studies (Alexandersen et al., 2020; van Kampen et al., 2020) described that detection of sgRNA outlived the detection of infectious virus. This presumably occurred because sgRNA is associated with cellular membranes and is nuclease resistant, which makes it stable or protects it from the host cell response (Van Hemert et al., 2008; Alexandersen et al., 2020). Therefore, the presence of sgRNA is not a direct evidence of active infection; instead, the presence of sgRNA at a lower level than that of genomic RNA results in its detection for a relatively shorter period of time (Alexandersen et al., 2020).

In accordance with our findings, previous studies analyzing the performance of various antigen assays reported a uniformly high specificity; however, a wide range of sensitivity was observed that seems to be less than the manufacturer’s reported range (Cerutti et al., 2020; Linares et al., 2020; Mak et al., 2020; Scohy et al., 2020). These differences can be caused by numerous factors, such as the testing time related to the phase of infection, sample size, site and quality, handling and preparation, or assessment of Ct-values using non-standardized rRT-PCR. The major strength of this study was the correlation of clinical samples in the cell culture for infectivity, which indicates the importance of Ag-RDTs in clinical practice. The limitations of the present study include the use of small sample size and the respiratory samples different from those recommended by the manufacturer. The use of previously stored samples, and the use of a modified method for processing samples in VTM; collectively, these factors may contribute to the degradation or dilution of the antigen.

The clinical sensitivity for the potential infectious samples and moderate to fair agreement with cell culture, as well as substantial to moderate agreement with rRT-PCR, allow large-scale application of Ag-RDT-based testing. Furthermore, effective screening and rapid results, in particular, depend on the testing frequency (Larremore et al., 2021). Due to the rapid turn-around time of the results, these assays can provide added value, for instance, in patients requiring emergency surgical procedure or in mass settings such as a long-term care facility, school, or workplace. Identification of individuals with positive results in 10–20 min allows earlier isolation and effective contact tracing compared to delayed results.

## Conclusion

Our results suggest that Ag-RDTs can effectively detect SARS-CoV-2-infected samples, particularly with moderate to high viral loads. Such point-of-care tests have the potential to improve public healthcare strategies for minimizing the spread of infection. Despite low analytical sensitivity, the tests are economical, and their frequent use on a large scale will serve as an important tool to suppress community transmission, particularly in conditions with limited access to molecular methods. Our findings suggest that SARS-CoV-2 Ag-RDT-based testing on a larger scale can be considered for detecting potentially infective individuals and limiting virus spread.

## Data Availability Statement

The raw data supporting the conclusions of this article will be made available by the authors, without undue reservation.

## Ethics Statement

The studies involving human participants were reviewed and approved by the Institutional Review Board (IRB) of Chosun University Hospital (CHOSUN 2020-04-003-002). The patients/participants provided their written informed consent to participate in this study.

## Author Contributions

MT: methodology, investigation, formal analysis, and writing original draft. D-MK: conceptualization, supervision, validation, and project administration. C-MK: editing and supervision. M-SB and YL: methodology and investigation. J-WS, NY, and DK: conceptualization and data curation. All authors contributed to the article and approved the submitted version.

## Conflict of Interest

The authors declare that the research was conducted in the absence of any commercial or financial relationships that could be construed as a potential conflict of interest.

## Publisher’s Note

All claims expressed in this article are solely those of the authors and do not necessarily represent those of their affiliated organizations, or those of the publisher, the editors and the reviewers. Any product that may be evaluated in this article, or claim that may be made by its manufacturer, is not guaranteed or endorsed by the publisher.
