# Supplementary material for: Viable Severe Acute Respiratory Syndrome Coronavirus 2 Isolates Exhibit Higher Correlation With Rapid Antigen Assays Than Subgenomic RNA or Genomic RNA
Source: Front Microbiol. 2021 Nov 12;12:718497. doi: 10.3389/fmicb.2021.718497 (PMC8633410; doi:10.3389/fmicb.2021.718497)
Supplement: Supplementary file 1 [file Data_Sheet_1.docx]

Supplementary Material

**Supplementary Figure 1.** **Probit analyses for detecting infectious virus in respiratory samples.** Cell culture **(a)** for the detection of subgenomic RNA **(b)** Ag rapid FIA positivity **(c),** and Ag Gold **(d)** with respect to days post symptom onset. Blue line represents the probit curve, and dotted red lines represent the 95% confidence interval. Circles indicate marker points.


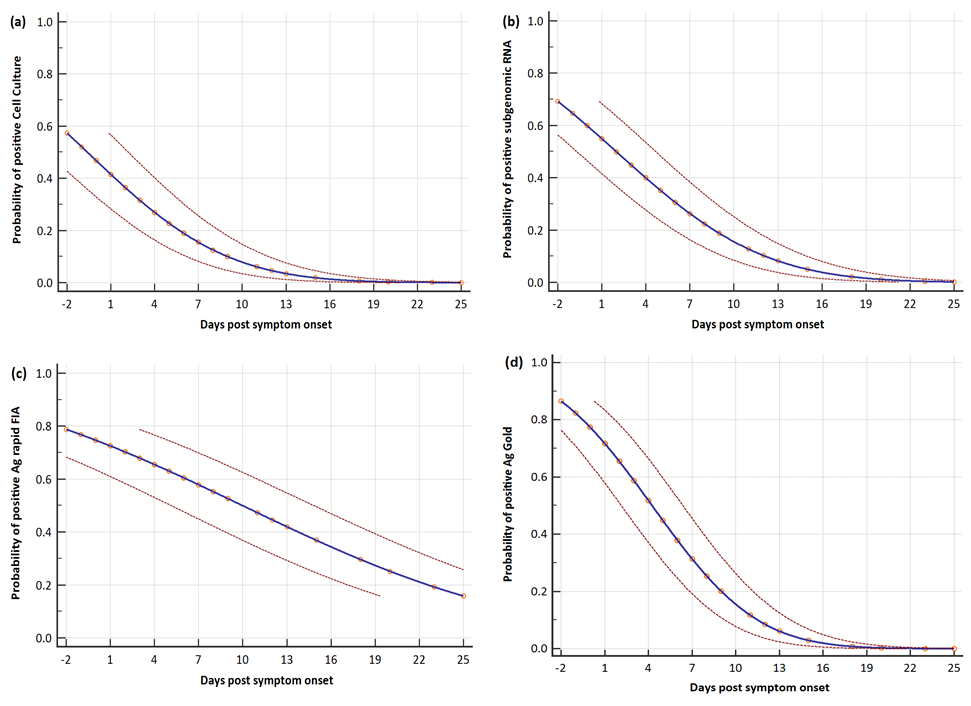


Days post symptom onset with less than 5% chances of positive results were: 11.7, 15.01, 34.60, and 13.56 days for SARS-CoV-2 Vero E6 culture, subgenomic RNA, Ag rapid FIA, and Ag Gold, respectively.

**Supplementary Figure 2:** **Assessment of subgenomic RNA in respiratory tract sample specimens.**


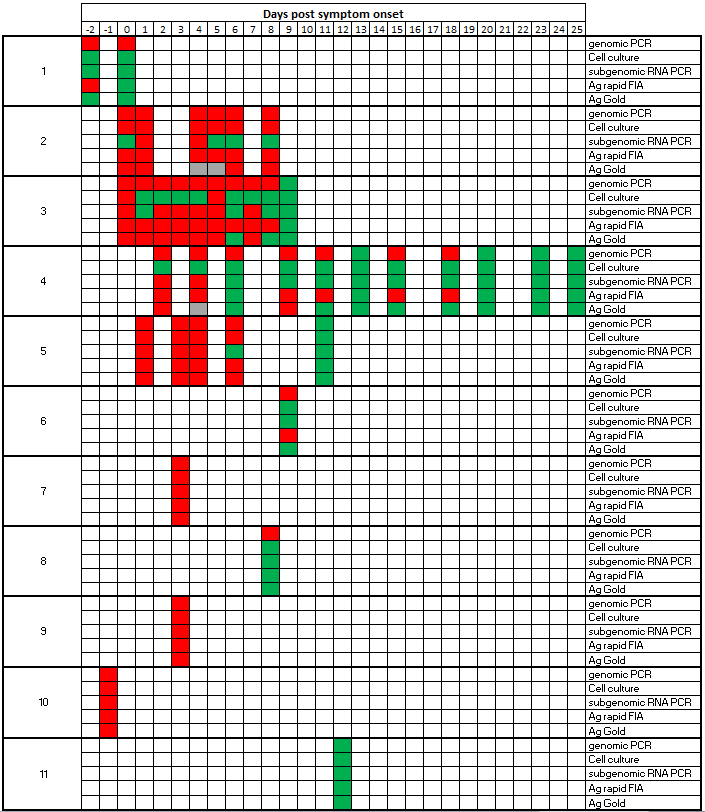


Individual patient charts with viral culture, Ag rapid FIA, and Ag Gold results in relation to the days post symptom onset. Positive test results are indicated in red, negative test results in green, and the unavailability of samples in grey. The assay was considered positive if any one or more respiratory samples (oropharynx, nasopharynx, or saliva) were positive on the same day as sample collection.

## Supplementary Table

Supplementary Table 1: SARS-CoV-2 RT-PCR, cell culture and SARS-CoV-2 Ag-RDTs results for the examined clinical samples.

| **Sample No.** | **Days PSO** | **SARS-CoV-2 rRT-PCR** | | | | **Cell Culture** | **Ag Rapid FIA** | **Ag Gold** |
| --- | --- | --- | --- | --- | --- | --- | --- | --- |
|  |  | **N-gene** | **E-gene** | **RdRp-gene** | **sgRNA** |  |  |  |
| 1 | -2 | 30.55 | 30.11 | 30.30 | UD | Neg | Pos | Neg |
| 2 | 0 | 31.64 | 31.74 | 32.21 | UD | Neg | Neg | Neg |
| 3 | 0 | 18.84 | 17.96 | 18.02 | UD | Pos | Pos | Pos |
| 4 | 1 | 17.85 | 17.74 | 17.88 | 24.06 | Pos | Pos | Pos |
| 5 | 1 | 36.53 | 34.42 | 34.64 | UD | Neg | Neg | Neg |
| 6 | 1 | 27.65 | 27.91 | 27.79 | UD | Neg | Pos | Pos |
| 7 | 4 | 27.47 | 27.54 | 27.69 | 32.70 | Neg | Pos | NA |
| 8 | 4 | 27.88 | 27.50 | 27.70 | 35.92 | Pos | Pos | NA |
| 9 | 5 | 31.44 | 31.83 | 31.91 | UD | Pos | Pos | NA |
| 10 | 5 | 28.00 | 27.50 | 28.37 | UD | Neg | Pos | NA |
| 11 | 6 | 26.48 | 25.40 | 25.55 | 31.45 | Pos | Pos | Pos |
| 12 | 6 | 29.35 | 29.24 | 29.39 | UD | Neg | Pos | Neg |
| 13 | 8 | 39.06 | UD | 39.06 | UD | Neg | Neg | Neg |
| 14 | 8 | 29.30 | 28.40 | 28.76 | UD | Pos | Pos | Pos |
| 15 | 0 | 20.70 | 21.27 | 21.48 | UD | Pos | Pos | Pos |
| 16 | 0 | 24.55 | 25.84 | 26.20 | 33.81 | Neg | Neg | Pos |
| 17 | 1 | 21.60 | 22.29 | 22.74 | UD | Neg | Pos | Pos |
| 18 | 2 | 24.77 | 25.57 | 25.80 | 31.36 | Neg | Pos | Pos |
| 19 | 3 | 25.05 | 26.14 | 26.01 | UD | Neg | Neg | NA |
| 20 | 3 | 23.36 | 24.24 | 24.35 | 30.53 | Neg | Pos | Pos |
| 21 | 4 | 23.92 | 24.91 | 24.87 | 29.75 | Neg | Pos | Pos |
| 22 | 4 | 28.21 | 29.38 | 29.14 | UD | Neg | Neg | Neg |
| 23 | 5 | 23.73 | 24.57 | 24.92 | 29.79 | Neg | Neg | Neg |
| 24 | 5 | 23.28 | 24.54 | 24.65 | 29.71 | Pos | Pos | Pos |
| 25 | 5 | 30.00 | 30.31 | 30.77 | UD | Neg | Pos | Neg |
| 26 | 6 | 29.95 | 31.06 | 31.30 | UD | Neg | Pos | Neg |
| 27 | 7 | 32.78 | 33.84 | 33.40 | UD | Neg | Neg | Neg |
| 28 | 7 | 23.65 | 24.61 | 24.42 | 30.55 | Neg | Pos | Pos |
| 29 | 7 | 22.46 | 23.84 | 24.33 | 28.24 | Neg | Pos | Pos |
| 30 | 8 | 32.62 | 33.39 | 34.35 | UD | Neg | Pos | Neg |
| 31 | 9 | 35.24 | UD | 37.95 | UD | Neg | Neg | Neg |
| 32 | 2 | 33.79 | NA | NA | UD | Neg | Neg | NA |
| 33 | 2 | 23.50 | NA | NA | UD | Neg | Pos | NA |
| 34 | 2 | 23.29 | NA | NA | 32.69 | Neg | Pos | Pos |
| 35 | 4 | 37.17 | NA | NA | UD | Neg | Neg | Neg |
| 36 | 4 | 23.67 | NA | NA | 32.89 | Neg | Pos | NA |
| 37 | 4 | 20.09 | NA | NA | 31.91 | Neg | Neg | Neg |
| 38 | 6 | 36.45 | NA | NA | UD | Neg | Neg | Neg |
| 39 | 6 | 29.01 | NA | NA | UD | Neg | Neg | Neg |
| 40 | 6 | 24.73 | NA | NA | UD | Neg | Neg | Neg |
| 41 | 9 | 35.38 | NA | NA | UD | Neg | Neg | NA |
| 42 | 9 | 26.96 | NA | NA | UD | Neg | Pos | Pos |
| 43 | 9 | 26.71 | NA | NA | UD | Neg | Pos | Neg |
| 44 | 11 | 33.67 | 35.77 | 36.60 | UD | Neg | Pos | Neg |
| 45 | 13 | 35.33 | NA | NA | UD | Neg | Neg | Neg |
| 46 | 15 | 32.12 | NA | NA | UD | Neg | Pos | Neg |
| 47 | 18 | 31.46 | NA | NA | UD | Neg | Pos | Neg |
| 48 | 23 | 37.10 | NA | NA | UD | Neg | Neg | Neg |
| 49 | 20 | 37.35 | NA | NA | UD | Neg | Neg | Neg |
| 50 | 25 | 36.46 | NA | NA | UD | Neg | Neg | Neg |
| 51 | 1 | 16.56 | 18.35 | 18.70 | 23.65 | Pos | Pos | Pos |
| 52 | 2 | 32.63 | 34.40 | 36.45 | UD | Neg | Neg | Neg |
| 53 | 3 | 19.10 | 20.30 | 20.40 | 24.15 | Pos | Pos | Pos |
| 54 | 4 | 22.27 | 24.21 | 24.40 | 29.33 | Pos | Pos | Pos |
| 55 | 6 | 24.99 | 26.92 | 27.24 | 32.66 | Neg | Neg | Neg |
| 56 | 6 | 32.31 | 35.00 | 34.51 | 31.2 | Pos | Pos | Pos |
| 57 | 11 | 37.27 | UD | UD | UD | Neg | Neg | Neg |
| 58 | 9 | 32.25 | 35.31 | 36.50 | UD | Neg | Pos | Neg |
| 59 | 3 | 25.01 | NA | NA | 32.64 | Pos | Pos | Pos |
| 60 | 8 | 34.47 | 31.18 | 30.43 | UD | Neg | Neg | Neg |
| 61 | 3 | 29.14 | NA | NA | 34.65 | Pos | Pos | Pos |
| 62 | -1 | 17.53 | 12.11 | 13.55 | 22.59 | Pos | Pos | Pos |
| 63 | 12 | 36.11 | 27.53 | 27.68 | UD | Neg | Neg | Neg |

Days PSO; days post symptom onset, sgRNA; subgenomicRNA
